# Temporal and Atemporal Provider Network Analysis in a Breast Cancer Cohort from an Academic Medical Center (USA)

**DOI:** 10.3390/informatics5030034

**Published:** 2018-08-06

**Authors:** Bryan D. Steitz, Mia A. Levy

**Affiliations:** 1 Department of Biomedical Informatics, Vanderbilt University, Nashville, TN 37201, USA; 2 Department of Medicine, Division of Hematology and Oncology, Vanderbilt University Medical Center, Nashville, TN 37201, USA

**Keywords:** social network analysis, clinical communication networks, clinical workflow

## Abstract

Social network analysis (SNA) is a quantitative approach to study relationships between individuals. Current SNA methods use static models of organizations, which simplify network dynamics. To better represent the dynamic nature of clinical care, we developed a temporal social network analysis model to better represent care temporality. We applied our model to appointment data from a single institution for early stage breast cancer patients. Our cohort of 4082 patients were treated by 2190 providers. Providers had 54,695 unique relationships when calculated using our temporal method, compared to 249,075 when calculated using the atemporal method. We found that traditional atemporal approaches to network modeling overestimate the number of provider-provider relationships and underestimate common network measures such as care density within a network. Social network analysis, when modeled accurately, is a powerful tool for organizational research within the healthcare domain.

## 1. Introduction

Social network analysis (SNA), applied in healthcare settings, has been used to understand provider communication [1–4], care team structures [5–7], knowledge sharing among clinicians [8–12], and the flow of patients between institutions [13–15]. SNA is an approach to study relationships between individuals. It explores hidden channels of collaboration and information flow among individuals and exposes potential disconnects in an organization [16–18]. SNA has been applied widely across technology, business, and manufacturing industries to identify trends [19–22] and improve efficiency [23–25]. However, SNA has been only minimally applied to healthcare domains. Secondary use of routinely collected health data, analyzed using SNA, can enable data-driven analysis at an organizational scale.

Provider interactions contribute to shared knowledge and effective patient management within healthcare organizations [13]. Both knowledge sharing and collaborative patient management are key features of multidisciplinary care [26–28]. Multidisciplinary care has received attention as an approach to deliver high-value care [29]. A study by O’Mahony and colleagues found that multidisciplinary inpatient rounding teams improved patient outcomes and reduced length of stay [30]. Similarly, a study by Kesson and colleagues discovered that multidisciplinary care was associated with improved survival in breast cancer patients [31]. Effective and timely communication is an important feature of multidisciplinary teams to maintain coordination of care [30]. Without appropriate coordination of care, patients experience treatment delays, higher costs, and poorer outcomes [32–34].

Social networks and inter-personal collaboration are inherently dynamic [35]. Networks evolve in response to new memberships and termination of existing relationships. Clinical networks change as new care patterns are adopted. Nonetheless, current methods of SNA use atemporal models of organizations [23]. These models simplify network dynamics and neglect the temporality of clinical care coordination [31]. To better apply SNA to healthcare contexts, it is necessary to devise a method that can more accurately represent dynamics of clinical care. In this study, we developed a temporal social network model to better represent care temporality. We apply our method to evaluate networks of clinicians treating stage I, stage II, and stage III breast cancer patients using outpatient appointment data collected from the electronic health record. We hypothesize that our method will better portray the patterns of clinical care compared to traditional, static, network analysis methods.

## 2. Materials and Methods

This study was conducted at the Vanderbilt-Ingram Cancer Center at the Vanderbilt University Medical Center, an academic tertiary care center located in middle Tennessee and a major referral center for the Southeastern United States. We collected outpatient appointment data from the electronic health record on patients who met inclusion criteria for the VUMC tumor registry; those who had been diagnosed or received part of the first course of their treatment at VUMC. The Vanderbilt University Institutional Review Board approved this study.

### 2.1. Study Population

We gathered data from the Vanderbilt University tumor registry on patients with stage I, stage II, or stage III breast cancer diagnosed between 1 January 2002 and 31 December 2016. The Vanderbilt University tumor registry collects cancer diagnosis and treatment data for all patients who were either diagnosed or received part of their first course of treatment at our institution [36,37]. Tumor registry data included a unique patient identifier, date of initial diagnosis, and cancer stage. We similarly extracted from the clinical data warehouse all respective appointment data two years prior to diagnosis date until 31 December 2016 for all patients included in our tumor registry cohort. Patients who had at least one outpatient visit with a provider between their date of diagnosis and six-months following their date of diagnosis were included in the study. Appointment data included a unique patient identifier, unique provider identifier, and appointment date. We mapped each unique provider to their national provider identifier (NPI) to determine their specialty [5]. For providers who were no longer practicing or did not have an NPI number, we used the role and specialty that was specified in the medical record.

### 2.2. Network Representation

To understand relationships among clinicians, we represented the data as a social network. Social networks consist of ‘nodes’, or entities that interconnect, and ‘edges’, which represent the existence of a relationship between entities. In our networks, nodes represent a clinician with whom a patient had an appointment. Edges represent a shared patient between two clinicians. Nodes and edges can additionally assume properties to further characterize relationships. In this network, the size of each node represents the total number of patients seen by a respective provider. The thickness of each edge represents the total number of patients shared between two providers.

**Figure 1.**
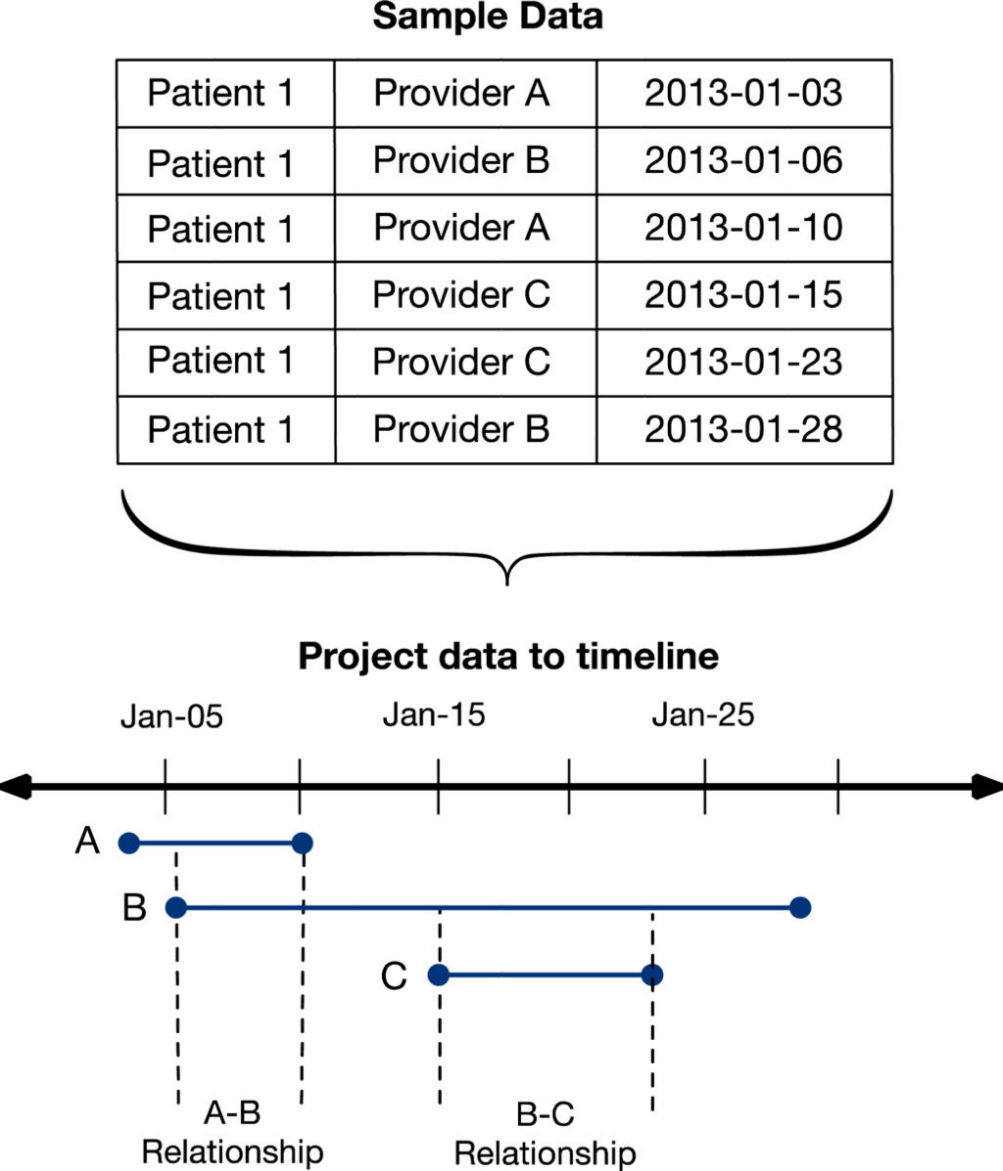
Temporal edge creation.

To create social networks, we extracted the set of all providers associated with an appointment for a patient in our cohort to define the list of nodes. We computed two types of edges: temporal and atemporal, such that we can compare network creation methods. To create temporal edges, we use a timeline projection approach to calculate provider pairs based on periods of overlapping care (Figure 1). We first obtain a list of all appointments for each patient, and sequence them in ascending order by appointment date. We iterate through the list of ordered appointments, recording the initial date that each provider entered the network as the ‘enter date’ and updating the last date in which each provider remained in the network as the ‘exit date’. Using the enter date and exit date for each provider, we examined overlapping time periods and calculated a relationship duration for each provider pair as the first and last dates that both providers were present in the network. To aggregate provider-pairs across patients, we take the sum of unique patients for whom the start date and end date of each provider-pair is included in a respective analysis timeframe.

To create atemporal edges we computed pairwise combinations of providers associated with care for a single patient such that each provider who treated a patient was paired with every other provider associated with a treatment of the same patient. We reduced edge combinations from our entire patient cohort to the set of unique relationships and an associated count of occurrences of the respective relationships.

### 2.3. Network Analysis

We analyzed social networks with respect to two types of temporality: absolute and relative. Absolute temporality refers to chronological time sequence, beginning from a specific date. Absolute temporal analyses assess how a network changes over time. Relative temporality refers to the difference in elapsed time between two events. Events occurring within a timeframe since diagnosis or since entering a network are analyzed in relative time. Respective to each type of temporality, we evaluate both institutional and provider networks. In our analysis, institutional networks refer to all providers and edges associated with the treatment of a patient in our cohort over a given time period; provider networks refer to the providers and edges connected to a single, central, provider who treated a patient in our cohort.

For each social network, we calculated the number of patients and providers included in the graph, and the respective number of relationships. Node and edge sizes were summarized with means, medians and ranges. We calculated descriptive statistics for the institutional network by year. Network measures are presented in Table 1. To assess network connectedness, we calculated yearly network density. We similarly calculated care density for each medical oncologist to quantify the amount of patient sharing among providers with whom the medical oncologist had a relationship [38]. Finally, we visualized each social network and assessed each node’s color to identify the significance of a particular specialty and collaboration between specialties. We created and visualized each network using the igraph [39] package within R 3.3.1 [40].

## 3. Results

Between 1 January 2002 and 31 December 2016, there were 6104 breast cancer patients included in the Vanderbilt University tumor registry, 5046 of whom had stage I, stage II, or stage III disease. We excluded 964 patients who did not have an outpatient visit with a provider between their initial diagnosis date and the following six months, restricting our analysis to 4082 patients. 2190 providers representing 68 unique specialties treated our patient cohort. Table 2 presents the outpatient provider network by stage. Stage I had the largest patient population and more provider-provider collaborations than either of the other stages. The number of shared patients between provider pairs was similar across all stages. Stage III patients saw, on average, more providers and had more appointments than either stage I or stage II patients.

Table 3 presents the institutional network statistics by year. In 2002, there were 155 new diagnoses, 456 providers, and 596 edges; fewer than any other year. There was a consistent yearly increase among all measures between 2002 and 2015. The number of shared patients (sum of edges) increased by 2794% between 2002 and 2015, the largest change across all measures in the institutional network. The institutional network density remained consistent across all studied years. There was the least growth (134%) in the number of unique providers treating our patient cohort. A total 39.8% of providers only entered the network for a single appointment with one patient, while 36.7% of providers remained in the network for at least one year. Providers remained, on average, in the network for 13.7 months with a median of 5.4 months. Oncology-related providers remained in the network for an average of 67 months with a median of 42.6 months; 42% of the oncology-related providers remained in the network for at least five years; 19.4% of oncology-related providers remained in the network for at least 10 years.

**Figure 2.**
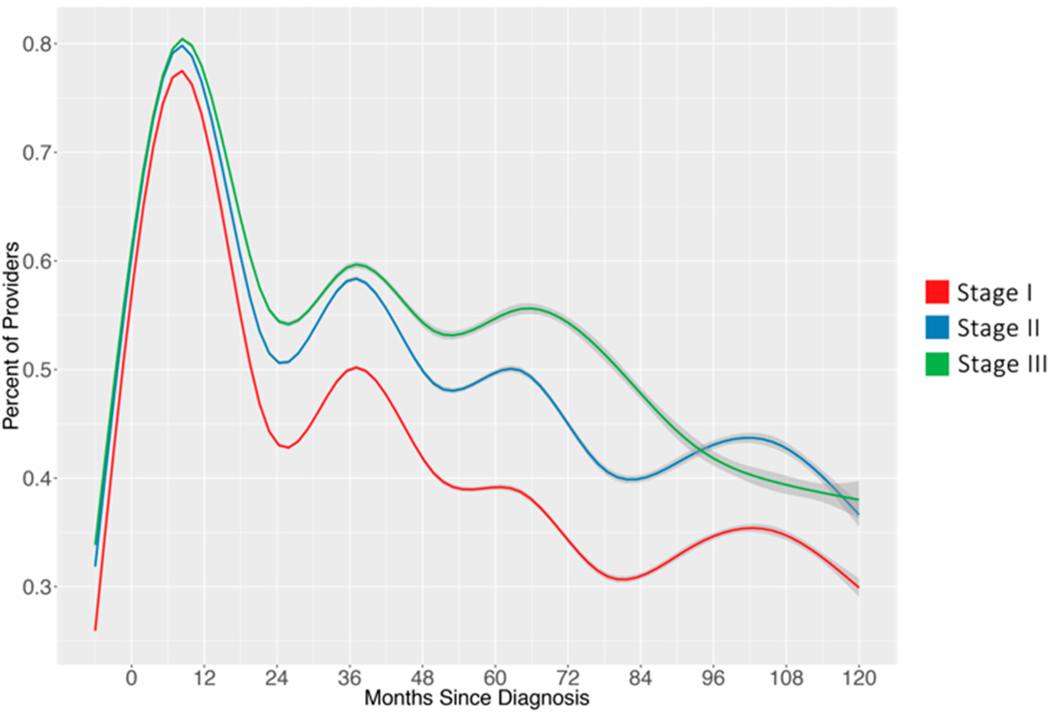
Percentage of appointments with oncology-related providers by month relative to diagnosis.

Between 2002 and 2015, there was a 170% and 165% growth in new breast cancer diagnoses and total patients, respectively. Nearly two-thirds (71.4%) of patients remain in the network for at least two years from their first appointment after diagnosis; 43% of possible patients remain in the network five years after diagnosis; while 14% of possible patients remain after 10 years. Figure 2 shows the percentage of oncology-related providers by stage and month relative to diagnosis date. Patients across all stages see the highest percent of oncology-related providers in the first year following diagnosis. For patients with stage II and stage III disease, oncology-related providers account for the majority of visits in the first five years following diagnosis.

**Figure 3.**
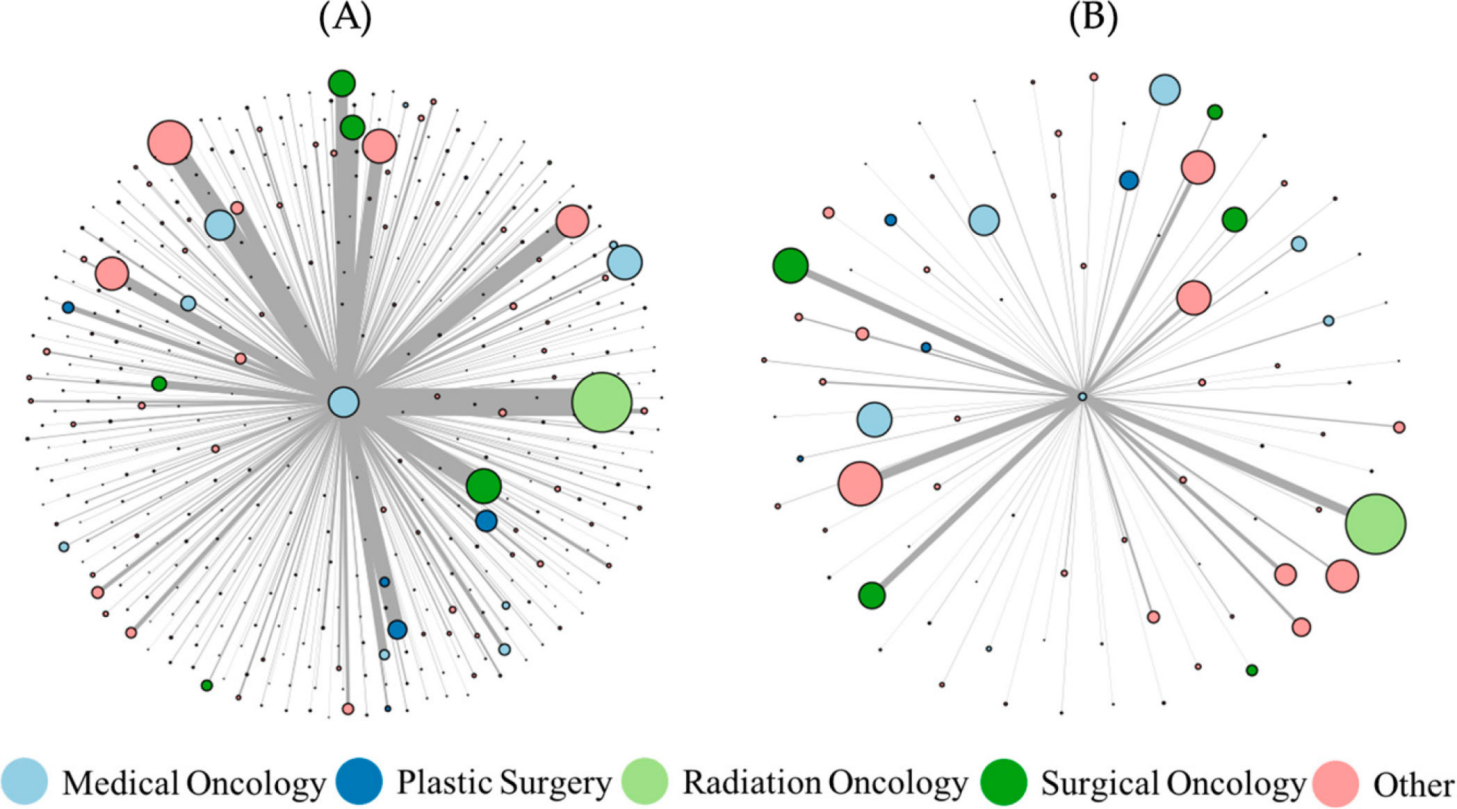
Care density for (**A**) top full-time provider and (**B**) top part-time provider by patient volume. The top full-time provider and top part-time provider are the central nodes in each respective graph. Edges are calculated using the temporal network creation method.

Table 4 presents the care densities for full-time and part-time medical oncology providers by year in network, relative to enter date. Care densities for the top (A) full-time and (B) part-time medical oncologist by patient volume are visualized in Figure 3. Full-time providers each have a higher care density than the part-time providers. The highest volume full-time provider treated 1155 patients, was connected to 1159 unique providers and had a care density of 12.3. Each of the full-time providers had more patients than provider-relationships, while part-time providers had more provider relationships than patients. The highest volume part-time provider treated 286 patients, was connected to 423 unique providers and had a care density of 6.9.

## 4. Discussion

This work makes contributions both to the field of network analysis and to the understanding of breast cancer care teams. This study advances the network analysis literature by presenting a temporal network model that is scalable throughout clinical environments using EHR data. We apply this model to understand care team composition for long-term cancer survivors in an academic medical center. Finally, this work contributes to the understanding of the work required of breast cancer providers to establish, maintain, and evolve a collaborative network of care team providers for their patients.

We have developed a temporal social network model to represent the dynamic collaborative relationships in clinical care using EHR appointment data for breast cancer patients. Using a timeline projection method for edge creation, we were able to represent providers entering and exiting the social network and assessed the evolution of collaborative relationships over time. Few prior studies have performed temporal social network analysis in the healthcare domain, but have relied on self-reported and observational data, rather than routinely collected health data, to model networks. A study by Samarth and colleagues surveyed clinicians in a pediatric acute care unit to analyze social networks for efficiency trends [41]. Other studies have modeled events sequentially to assess temporal relationships [42–44]. Chen and colleagues developed a model to discover bundled care opportunities by sequentially modeling events from the EHR [43]. Other prior studies have relied on dynamic analyses to assess dispersion phenomena [45,46]. One study by Christakis and Fowler examined the influence of individuals in the Framingham study dataset [47].

Our methodology offers a scalable approach to analyze provider networks within a single institution. The scalable approach is supported by the use of EHR appointment data. EHR data sources allow us to evaluate a broad range of providers, extending the breadth of single payer data across a single institution. In our prior work, we used VUMC tumor registry data to evaluate networks between cancer providers both inside and outside of our healthcare delivery system [5]. Use of EHR data similarly extends the breadth of providers such that we can evaluate ancillary providers who are integral to the cancer care team but not directly involved in cancer care. Furthermore, the use of appointment data allows us to evaluate the number of encounters between a patient and a provider rather than only the existence of a relationship. Incorporating encounter frequency allows us to evaluate provider collaboration by their relevance to patient care.

To our knowledge, this study is one of the first to use data from the electronic health record to temporally assess provider networks. A comparison of atemporal and temporal edge creation methods indicated that the traditional atemporal method of edge creation greatly over estimates the number of relationships between providers in the network. The accurate representation of edges has important implications for existing network analysis research [48]. Across our entire network, there were 249,075 atemporal and 54,695 temporal edges. Similarly, provider degree centrality in the temporal network was nearly half the atemporal degree centrality. Our method of edge creation more accurately reflects patterns of clinical care in that providers who treat a patient over the same time period likely coordinate actively through clinical messaging or conversation, or passively, through reading provider notes from a similar treatment period.

Our scalable approach is not without limitations. Our data was limited to appointments at a single institution and may not fully represent the patient’s entire scope of care that occurs at outside institutions. Payer data may better reflect a patient’s full scope of care across institutions, however with a large number of payers in our system, the data is difficult to acquire across an entire population. We could improve our networks by incorporating additional data sources. Wang and colleagues incorporated billing data to model social networks [49]. Future studies could incorporate billing data, clinical communications between providers, electronic whiteboard data [50], clinical documentation, orders, and other EHR artifacts to better represent an institution’s entire social network.

This study is one of the first to address temporal changes in networks. We looked at institutional networks and provider networks in relative and absolute time, which attempts to assess the evolution of care networks at a low level. Our results from the institutional network analysis indicated that the number of patients treated for breast cancer more than doubled over our studied period. A similar growth in yearly diagnoses contributed to an increasing patient population. We attribute this growth to an increase in the regional population surrounding our medical center and the growing positive reputation of our comprehensive cancer center. This also demonstrates the impact of long-term survivors of breast cancer treatments in that they maintain relationships with their oncology care team for a lengthy period of time. There were 43% and 14% of patients still in the network after 5 years and 10 years, respectively. We expect that some of these patients are on adjuvant hormone therapy, which often continues for five to ten years following diagnosis. However, in other secondary analyses, we found that many of these patients are receiving subsequent, non-cancer related, treatments at our medical center. Of those patients still in the network at 5 and 10 years, cancer providers made up only 47% and 32% of their care teams. We hypothesize that the cancer treatments introduce a ‘medical home’ phenomenon, in which patients who are already receiving care at our institution will similarly receive care for additional, non-cancer related health conditions. These data could inform optimal care team composition and resource allocation for long-term management of cancer survivors within a medical center.

Our absolute time analysis of the institutional network indicated that the number providers more than doubled while the number of edges increased more than 2400% over our studied period. Despite this growth, network density remained relatively stable by year, indicating that providers maintain a high degree of connectivity in cancer patient care coordination despite colleagues joining and leaving the network. In our relative time provider network analysis, we were able to identify a considerable difference in care densities between full-time and part-time medical oncologists. Full-time medical oncologists had a relatively stable care density over time, while the care density of part-time medical oncologists increased yearly. We hypothesize that full-time providers establish members of their care team more quickly than part-time providers. Nonetheless, all medical oncologists had an increase in care density after the first year, indicating a startup period in which each provider becomes established in their network. Network density reflects the work a provider must do to establish, maintain, and evolve care coordination collaborations among their provider peers. Once established, the density of the medical oncology provider network remained relatively stable over time. The composition of the members of that network was highly dynamic, representing a continuous effort to establish and maintain new relationships with other providers.

## 5. Conclusions

Social network analysis, when modeled accurately, is a powerful tool for organizational research within the healthcare domain. While early data suggests that providers who are more tightly connected may have better clinical outcomes and lower costs, few formal methods exist to accurately model networks over time. Current methods utilize single payer claims data and rely on pairwise provider combinations to model connectivity. We employed a timeline projection approach to edge creation. We found that traditional atemporal approaches to edge creation overestimate the number of provider-provider relationships and underestimate measures such as care density within the network. Applying social network analysis to our temporal approach to edge creation can promote quantitative approaches to more accurately describe complex provider care networks that can be used to evaluate care coordination and correlation with clinical outcomes. Future applications of this modeling strategy will be used to understand how provider connectivity relates to treatment outcomes and to assess the relationship between provider connectivity and communication patterns to understand operational efficiency.

## Figures and Tables

**Table 1.** Network analysis measures.

	Calculation	Definition	Interpretation
Network Density	*2(Number o f Edges)*	The percentage of potential connections in a network that are actual connections.	A measure to quantify the relative degree of connectivity within a network.
	*(Number of Nodes)(Number of Nodes−1)*		
Network Care Density	*Sum of Edge Weights*	The average number of patients shared per provider connection.	A measure to quantify the amount of patient sharing between providers in a network.
	*Total Number o f Edges*		
Degree Centrality	The sum of unique connections adjacent to a single node.	The total number of connections associated with a single node.	The number of providers who share a patient with a single provider of interest.
Atemporal Edge	Pairwise combination of providers associated with treatment of a single patient.	Connection between nodes, irrespective of time when node was present in the network.	Provider–provider connections that represent potential connections based on caring for a shared patient.

**Table 2.** Outpatient network statistics by cancer stage.

	Stage I	Stage II	Stage III	Stage I–III
Number of Patients	2116	1452	514	4082
Number of Providers	1090	948	503	2190
Unique Temporal Edges	35,402	23,265	9789	54,695
Unique Atemporal Edges	167,318	107,018	41,686	249,075
*Node Size*				
Mean (range)	16.3 (1, 1084)	10.7 (1, 675)	5.6 (1, 164)	31.4 (1, 2351)
Median	4	3	2	179
*Temporal Edge Size*				
Mean (range)	3.4 (1, 371)	3.5 (1, 400)	3.1 (1, 164)	4.2 (1, 838)
Median	2	2	2	2
*Atemporal Edge Size*				
Mean (range)	1.8 (1, 467)	1.7 (1, 306)	1.5 (1, 157)	2.2 (1, 908)
Median	1	1	1	1
*Providers per Patient*				
Mean (range)	15.3 (1, 414)	15.1 (1, 64)	15.7 (1, 44)	15.3 (1, 74)
Median	12	12	13	12
*Appointments per Patient*				
Mean (range)	70.5 (1, 414)	72.8 (1, 498)	80.4 (1, 363)	72.7 (1, 498)
Median	50	56	65	54

**Table 3.** Institutional network statistics by year.

	Number of Diagnoses	Number of Patients	Number of Providers	Number of Temporal Edges	Number of Atemporal Edges	Sum of Temporal Edge Weights	Sum of Atemporal Edge Weights	Temporal Network Density	Atemporal Network Density

2002	155	1424	458	596	2033	1814	2831	1.56	1.57
2003	156	1678	533	1309	3355	4425	4843	1.52	1.62
2004	174	1840	569	1919	4273	6575	6239	1.7	1.76
2005	173	2023	631	2748	5095	9714	7550	1.73	1.7
2006	202	2249	682	3378	5340	12,082	8078	1.69	1.53
2007	205	2461	753	4372	6625	15,408	9993	1.63	1.6
2008	256	2625	786	5455	7962	18,805	12,177	1.66	1.63
2009	276	2799	790	7055	9453	24,153	14,800	1.85	1.79
2010	271	2989	863	9658	11,841	33,066	19,188	1.88	1.94
2011	303	3127	945	11,581	13,614	40,552	22,146	1.82	1.84
2012	331	3366	995	13,016	14,601	44,778	23,394	1.8	1.69
2013	406	3593	1038	14,663	16,387	51,366	27,015	1.84	1.74
2014	356	3711	1034	14,729	16,212	52,382	26,794	1.83	1.83
2015	418	3775	1074	15,366	17,493	52,505	28,240	1.66	1.76
2016	400	3826	1076	14,142	17,025	49,263	29,263	1.5	1.74

**Table 4.** Medical oncologist care densities, relative to date at which each provider entered the network.

		Full-Time			Part-Time	
	Medical Oncologist 1	Medical Oncologist 2	Medical Oncologist 3	Medical Oncologist 4	Medical Oncologist 5	Medical Oncologist 6
*Overall Degree Centrality*						
Temporal	1159	1034	950	423	517	342
Atemporal	1963	1979	1864	994	1493	836
*Overall Care Density*						
Temporal	12.3	12.3	14.2	6.9	7.8	5.4
Atemporal	7.2	6.4	7.2	2.9	2.7	2.2
*Yearly Temporal Care Density*						
Year 1	4.1	4.86	6.4	3.77	2	3.74
Year 2	6.64	6.69	7.07	4.49	4	4.11
Year 3	5.95	7.72	6.96	4.47	5.04	4.34
Year 4	6.94	7.69	7.6	5.11	5.16	4.2
Year 5	6.76	7.51	8.79	4.97	5.84	4.22
Year 6	6.69	7.46	9.34	5.03	6.19	
Year 7	6.76	6.86	9.33	5.49		
Year 8	7.38	7.23	9.36			
Year 9	7.64	7.24				
Year 10	7.17	7.28				
Year 11	7.23	6.74				
Year 12	7.25					
Year 13	6.68					
Year 14	6.66					
